# Melatonin promotes goat spermatogonia stem cells (SSCs) proliferation by stimulating glial cell line-derived neurotrophic factor (GDNF) production in Sertoli cells

**DOI:** 10.18632/oncotarget.12720

**Published:** 2016-10-18

**Authors:** Bowen Niu, Bo Li, Chongyang Wu, Jiang Wu, Yuan Yan, Rui Shang, Chunling Bai, Guangpeng Li, Jinlian Hua

**Affiliations:** ^1^ College of Veterinary Medicine, Shaanxi Stem Cell Engineering and Technology Research Center, Northwest A&F University, Yangling 712100, Shaanxi, China; ^2^ College of Agriculture, Guangdong Ocean University, Zhanjiang 524088, China; ^3^ Key Laboratory for Mammalian Reproductive Biology and Biotechnology, Ministry of Education, Inner Mongolia University, Hohhot 010021, China

**Keywords:** melatonin, spermatogonia stem cells (SSCs), GDNF, Sertoli cells, proliferation

## Abstract

Melatonin has been reported to be an important endogenous hormone for regulating neurogenesis, immunityand the biological clock. Recently, the effects of melatonin on neural stem cells (NSCs), mesenchymal stem cells(MSCs), and induced pluripotent stem cells(iPSCs) have been reported; however, the effects of melatonin on spermatogonia stem cells (SSCs) are not clear. Here, 1μM and 1nM melatonin was added to medium when goat SSCs were cultured *in vitro*, the results showed that melatonin could increase the formation and size of SSC colonies. Real-time quantitative PCR (QRT-PCR) and western blot analysis showed that the expression levels of SSC proliferation and self-renewal markers were up-regulated. Meanwhile, QRT-PCR results showed that melatonin inhibit the mRNA expression level of SSC differentiation markers. ELISA analysis showed an obvious increase in the concentration of GDNF (a niche factor secreted by Sertoli cells) in the medium when treated with melatonin. Meanwhile, the phosphorylation level of AKT, a downstream of GDNF-GFRa1-RET pathway was activated. In conclusion, melatonin promotes goat SSC proliferation by stimulating GDNF production in Sertoli cells.

## INTRODUCTION

Melatonin has a series of functions, such as acting as an antioxidant, regulating the immune system, and as an anti-tumor factor [1]. The pineal gland is an important endocrine organ, it can secrete melatonin, and the concentration secreted is dependent on the photoperiod in mammals [2–4]. For instance, psychopathology scores were significantly and negatively correlated with melatonin production in the summer and winter [5], and the reproduction of Chinese Guanzhong dairy goat was also seasonal. Some studies showed that the reproductive seasonality of subtropical goats was mainly controlled by changes in the photoperiod [6, 7], so whether melatonin had a relationship with seasonal reproduction is unclear in male goats.

Induced pluripotent stem cells (iPSCs) is obtained when inducing the somatic cells by defined factors *in vitro*, it has the same function as embryonic stem cells (ESCs), while it has very a low reprogramming efficiency in the process of inducing. Studies have shown evidences that melatonin can enhance the production of iPSCs due to its antioxidant activity [8, 9]. In human adult MSCs, for instance, melatonin combined with the extracellular matrix can enhance cellular alkaline phosphatase (AP) activity, induce osteogenesis via the melatonin receptor 2 (MT2), and also reverse stress-induced MSC injuries [10–13]. Meanwhile, melatonin can elevate the viability of rat neural stem cells (NSCs) and induce NSCs differentiate into dopaminergic neurons and decrease astrocyte production [14, 15]. Interestingly, some studies have shown that melatonin can increase the production of Brain-Derived Neurotrophic Factor (BDNF) and glial cell-derived neurotrophic factor (GDNF) in cultured NSCs, both of which are integral to neuronal development and differentiation [16]. As the only adult stem cells that can transfer genetic information to the next generation, spermatogonia stem cells (SSCs) would be an important model for studying infertility, evolution, mammalian breeding and so on. However, as an important regulator of animal reproduction, the effect of melatonin on SSCs remains obscure.

SSCs are located at the basal membrane of the seminiferous tubule in the testes. The microenvironment where SSCs reside, termed as a niche, could maintain SSC population stability by proliferation, self-renewal and differentiation into mature spermatids [17, 18]. GDNF has been reported to be an essential niche molecule for regulating the fate of undifferentiated spermatogonia [19]. GDNF can bind to its ligand-specific co-receptor, GDNF family receptor alpha-1 (GFRA1), which is a membrane protein that is expressed in most spermatogonial subtypes in mouse testes including As, Apr, and Aal spermatogonia, triggering signaling via the transmembrane receptor tyrosine kinase RET [20–22]. The GDNF-GFRa1-RET complex can activate PI3K (phosphoinositide 3-kinase) intercellular signaling mechanisms and lead to downstream activation of Akt, which then influences the survival and proliferation of SSCs [23].

In this study, we investigated the effects of melatonin on dairy goat SSCs cultured *in vitro*. We found that melatonin could promote the formation of goat SSC colonies and enhance the proliferation and self-renewal ability of dairy goat SSCs. Meanwhile, Enzyme-linked immunosorbent assay (ELISA) results showed that melatonin could stimulate Sertoli cells producing GDNF. Then the increased phosphorylation of AKT further showed melatonin promoted goat SSC self-renewal and proliferation through the GDNF-GFRa1-RET-AKT pathway.

## RESULTS

### Spermatogenesis in goat testis was seasonal

**Figure 1:**
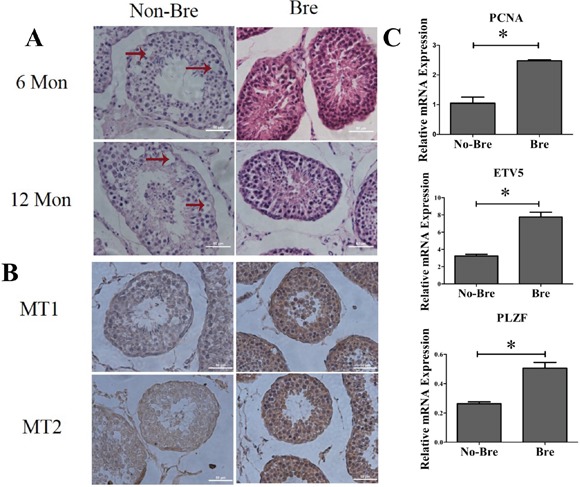
Spermatogenesis in goat testis was seasonal **A.** Hematoxylin-eosin staining analysis of the different ages of goat testis during the breeding and non-breeding seasons, Mon: month. Arrow: vacuolationin the seminiferous tubule. Bre: breeding season. No-Bre: non-breeding season. **B.** MT1 and MT2 immunohistochemistry staining of 1year age goat testisslice. MT: melatonin receptor. **C.** QRT-PCR analysis of the markers for proliferation and self-renewal.*: P<0.05.

To verify the difference in spermatogenesis in the breeding and non-breeding seasons, we collected 6 pairs of goat testes samples at 6 month and 12 month in April and December, respectively. The hematoxylin-eosin staining of seminiferous tubule showed that during the breeding season, the spermatogenesis related cells, including spermatogonia, spermatocytes and spermatids were densely arranged. During then on-breeding season, the cells were distributed loosely and appeared vacuolation (as indicated by the arrows) due to low spermatogenesis levels (Figure 1A). As the secretion of melatonin by the pineal gland is controlled by illumination intensity and the light intensity during the breeding season was low, which reminded us melatonin may affect spermatogenesis. We tested the expression of melatonin receptors MT1 and MT2 in 1-year old goat testis using immunohistochemistry, and the results showed that MT1 and MT2 were expressed in almost every stage of spermatogenesis and both of the receptors were more highly expressed during the breeding season than the non-breeding season (Figure 1B). Meanwhile, QRT-PCR analysis of the whole testis fragments showed that the expression of the SSC self-renewal markers Plzf and Etv5 were approximately 2-fold higher during the breeding season than the non-breeding season and that the expression of cell proliferation marker Pcna during the breeding season was 2.5-fold higher than the non-breeding season (Figure 1C). These results showed that spermatogenesis in goat testis was seasonal and that melatonin plays an important role in the regulation of dairy goat spermatogenesis.

### Melatonin promoted proliferation and inhibit differentiation of goat SSCs

**Figure 2:**
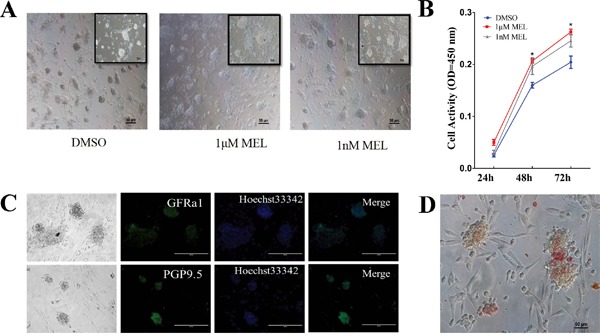
Melatonin can promote goat SSCs colony formation **A.** Light field of SSCs colony cultured with melatonin, bar=50 μm. **B.** CCK-8 analysis of cultured SSCs activity. *: P<0.05. **C.** Immunofluorescence staining of goat SSCs colonies, bar=200 μm. **D.** AP staining of goat SSCs, bar=50 μm.

To determine whether melatonin can affect the SSCs culture *in vitro*, we separated and purified the spermatogonia using differential attachment techniques and cultured the cells using SSC medium containing different concentrations of melatonin. The results showed that melatonin could increase the formation and size of SSC clusters (Figure 2A). A CCK-8 assay showed that the cell viability and multiplication rate in the group with melatonin addition was approximately 23% higher than those of the control (DMSO) group at 48 h. The group in which 1 μM of melatonin was added also had a higher multiplication rate than the other two groups at 72 h (Figure 2B). To verify that the cells that we cultured were SSCs, we stained the cultured cells with SSC markers GFRa1 and PGP9.5 using immunofluorescence staining. The results showed that the cell colonies were positive for GFRa1 and PGP9.5 (Figure 2C). The AP staining also indicated that the cells were AP positive and that the cultured cells were putative SSCs (Figure 2D).

**Figure 3:**
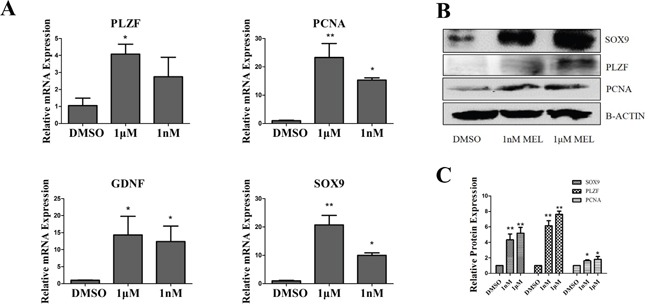
Analysis of goat SSCs and Sertoli cell makers **A.** QRT-PCR and western blot analysis of proliferation, self-renewal and Sertoli cell markers. **B.** Western blot analysis of proliferation, self-renewal and Sertoli cell markers. **C.** Quantification of western blot *, P<0.05,**, P<0.01.

To confirm the effects of melatonin on goat SSC proliferation, we tested the mRNA and protein expression level changes. The results of QRT-PCR showed that the SSC proliferation marker PCNA increased ~15-22-fold and the self-renewal marker PLZF increased ~3-4-fold in the melatonin added group (Figure 3A). Western blotting showed that PCNA increased approximately 2-fold and PLZF increased ~6-8-fold in the melatonin added group (Figure 3B). This indicated that melatonin could promote cell proliferation and enhance the SSC self-renewal ability. The cells separated from goat testis included Sertoli cells, which can secrete GDNF. We tested the Sertoli cell markers SOX9 and GDNF. Interestingly, GDNF and Sox9 mRNA were ~12-14-fold and ~10-20-fold up-regulated, respectively. When 1 nM and 1 μM melatonin were added, and the protein SOX9 was also increased in the melatonin treatment group (Figure 3A, 3B, 3C).

**Figure 4:**
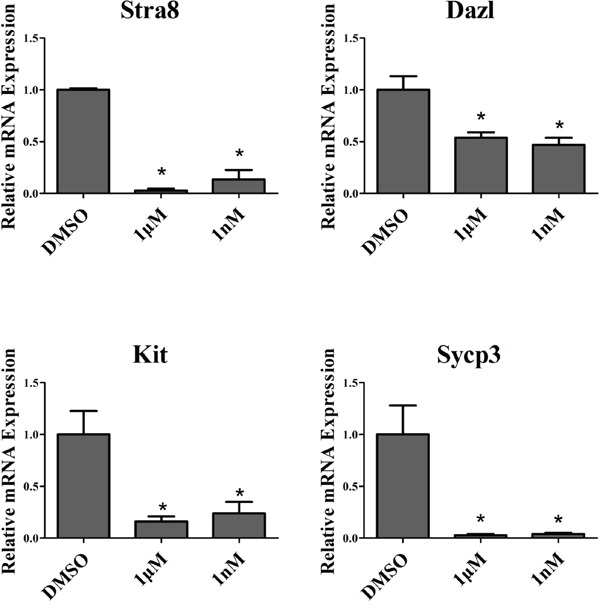
QRT-PCR analysis of dairy goat SSC differentiation markers. *, P<0.05

Stra8 can regulate the meiotic initiation in both spermatogenesis and oogenesis [28]. Dazl was an RNA binding protein that required for spermatocyte meiosis, the over-expression of Dazl promoted the expression of meiosis-related genes in dairy goat mGSCs [29]. Kit was the transmembrane tyrosine kinase receptor for stem cell factor, spermatogonia could not differentiate without kit presence [30]. Synaptonemal complex protein SYCP3 play an important role during the meiotic, the mutations of SYCP3 can cause meiotic arrest [31]. To confirm the effect of melatonin on goat SSC differentiation, we tested the mRNA expression of Stra8, Dazl, Kit and Sycp3, which all of them were markers of goat SSC differentiation. The results showed that the mRNA expression level all were decreased, both in 1 nM and 1 μM melatonin, compared with the DMSO group (Figure 4).

### Melatonin regulated the proliferation of dairy goat SSCs through GDNF

**Figure 5:**
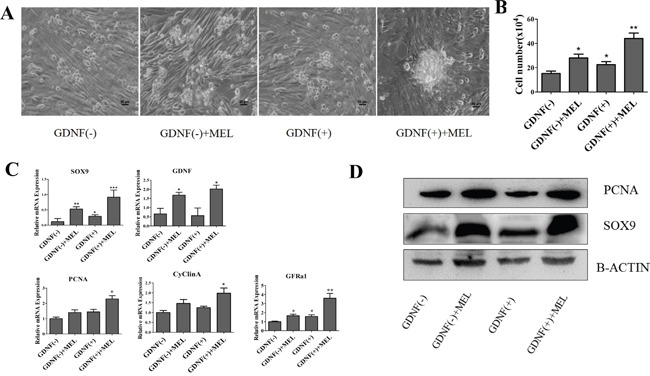
Analysis of the effect of GDNF and melatonin on goat SSCs culture **A.** Light field of SSCs cultured with melatonin and GDNF, bar=50 μm. **B.** Cell density after being cultured with different cell mediums at 48 h. The initial number was 5*10^4^. **C.** QRT-PCR and western blot analysis of proliferation, self-renewal and Sertoli cell markers. **D.** Western blot analysis of proliferation and Sertoli cell markers. *, P<0.05,**, P<0.01.

To study whether melatonin effect the proliferation of goat SSCs directly, we treat mGSCs-I-SB cell, which was a CD49f-positive cell, with melatonin and analysis the activity of cells. Interestingly, we found melatonin has not significant effects on the proliferation of mGSCs-I-SB cell (Supplementary Figure S1 and S1 Text). As described in Figure 3, melatonin increased the expression of GDNF. We cultured the purified testicular cells in four groups: SSC culture medium without GDNF (GDNF(−)), SSC culture medium (GDNF(+)), SSC culture medium without GDNF with melatonin added (GDNF(−)+MEL), and SSC culture medium with melatonin added (GDNF(+)+MEL). The results showed that the SSC colonies formed in the melatonin added medium were larger, SSC colonies in group GDNF(+)+MEL were larger than those in group GDNF(+), and group GDNF(−)+MEL was larger than group GDFN(−) (Figure 5A). This indicated that GDNF was essential for SSC culture *in vitro*. We then counted the number of cells. After treating the cells for 48 h, the number of cells in group GDNF(+)+MEL were approximately 2-fold higher than those in group GDNF(+) and group GDNF(−)+MEL was approximately 1.8-fold higher than group GDNF(−) (Figure 5B). In the QRT-PCR assay, we found that the mRNA expression of Gfra1, a specific receptor of GDNF in SSCs; proliferation marker Pcna; and cell cycle protein CyclinA was higher in the melatonin or GDNF added media than in the GDNF(−) group (Figure 5C). The expression of Sertoli cell markers Sox9 and GDNF was higher in the GDNF(−)+MEL and GDNF(+)+MEL groups than in the GDNF(−) and GDNF(+) groups (Figure 5C). The western blot results also showed that the expression of PCNA and SOX9 was increased in the GDNF (−)+MEL and GDNF (+)+MEL groups (Figure 5D).

**Figure 6:**
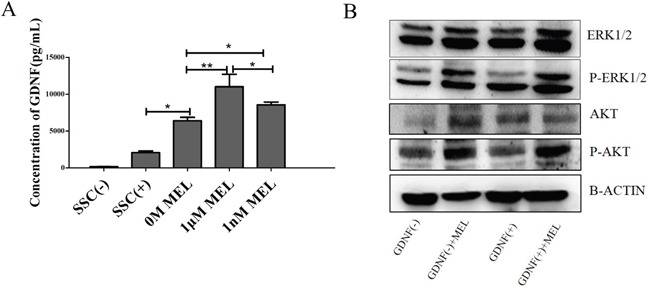
Concentration of GDNF in SSCs medium and the pathway of melatonin affect **A.** ELISA analysis of GDNF levels in the SSCs medium. **B.** Western Blot analysis of phosphorylation levels of AKT and ERK. *, P<0.05,**, P<0.01.

To ensure that melatonin can stimulate GDNF production during SSC culture, we collected the cell culture medium after treating SSCs with 0 M, 1 μM and 1 nM of melatonin for 48 h. We analyzed the concentration of GDNF using a GDNF ELISA kit. The SSC medium (SSCs(+)) and SSC medium without GDNF (SSC (−)) were used as positive and negative controls. The results showed that the concentration of GDNF in the 1 μM MEL group increased the most compared with the other group, followed by the 1 nM MEL group. The results also showed that in the 0 M MEL added group, the GDNF concentration was enhanced approximately 3-folds compared with that of the SSC (+) group, indicating that Sertoli cells can secrete GDNF in the culture of goat SSCs *in vitro* and that melatonin can stimulate Sertoli cells to produce more GDNF (Figure 6A). Because GDNF-GFRa1-RET mediated SSC self-renewal and proliferation pathways have been reported over the past several years [32], we analyzed the phosphorylation levels of AKT, which is the downstream GDNF-GFRa1-RET pathway, using western blotting. The results showed that the phosphorylation levels of AKT were higher in the GDNF(−)+MEL and GDNF(+)+MEL groups than in the other groups. Meanwhile, the phosphorylation levels of ERK in the GDNF(+)+MEL group was higher than the other three groups, with the GDNF(−) group demonstrating the lowest phosphorylation levels (Figure 6B). This study further indicated that the activation phosphorylation levels of AKT maybe need both GDNF and MEL, and the accelerated proliferation of goat SSCs by melatonin was through the GDNF-GFRa1-RET mediated SSC self-renewal and proliferation pathway.

## DISCUSSION

Melatonin is an important factor for regulating sleep, immunity and even aging and is an essential regulator for mammal reproduction [33, 34]. In our study, we found that the melatonin receptors MT1 and MT2 in the goat seminiferous tubule were increased during the breeding season, indicating that melatonin during the breeding season increased and influenced the process of spermatogenesis. Meanwhile, many studies have shown that melatonin receptors are expressed in spermatozoa and spermatocytes [35–37]. However, we found that melatonin receptors are expressed in almost every subtype of spermatogenesis cells in dairy goats. The tight spermatogenesis during the breeding season indicated that the proliferation of spermatogenic cells increased, including SSCs. Because of the complex spermatogenesis regulation network *in vivo*, we analyzed the relationship between melatonin and SSCs *in vitro.*

CD49f is a significant cell-surface marker for SSC and it can be used in the magnetic separation of SSCs. In our study, melatonin had none effect of the proliferation of an immortalized CD49f positive dairy goat male germline stem cells (mGSCs-I-SB) [38]. However, we found that melatonin can promote the primary SSC colony formation and promote the proliferation markers expression, this indicated that melatonin may affect goat SSCs indirectly. Interestingly, we also found that melatonin can inhibit the expression of SSC differentiation markers expression, which was contrary to the recently report that melatonin can promote the development of sperm from early developing spermatogenic cells of Suffolk sheep [39]. Here, we speculated melatonin may function though different ways to regulate SSCs when cultured in different medium.

In the testes, Sertoli cells are located close to the spermatogonia and supplied a suitable niche for spermatogenesis [40]. Sertoli cells can secrete several factors, such as GDNF, fibroblast growth factor-2 (FGF2), bone morphogenetic protein 4 (BMP4) and so on, all of which are related to spermatogenesis [36]. Recent reports have shown that exogenous melatonin acts via its receptors and appears to play regulatory roles in the development and function of bovine Sertoli cells [37]. In our study, ELISA showed that the GDNF concentration was increased in the melatonin added group, indicating that melatonin can promote Sertoli cell produced GDNF.

**Figure 7:**
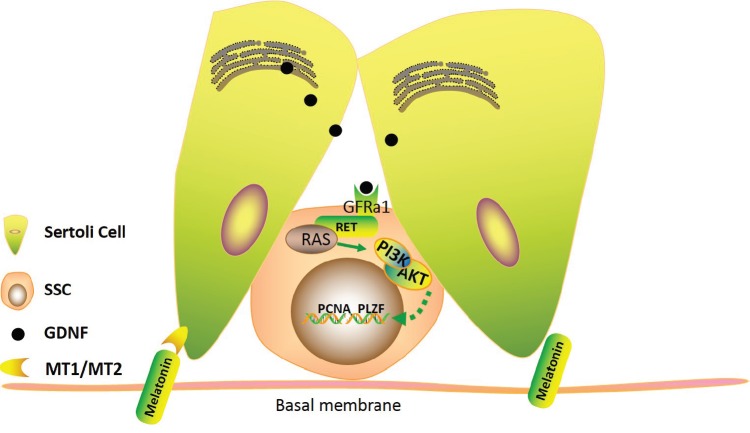
Model for the effect of melatonin on dairy goat SSC proliferation

GDNF is a distant member of the transforming growth factor–β (TGF–β) family and is an important neurotrophic factor in the regulation of the survival and differentiation of neuronal cells [41]. Spermatogenesis in heterozygous GDNF knockout mice gradually disappears and leads to infertility. In contrast, the A_pr_ and A_al_ spermatogoniain in the GDNF overexpressed mice accumulated in the seminiferous tubules [42]. Each Gdnf-, Gfra1- and Ret-deficient mouse showed severe SSC depletion and infertility. GDNF-mediated RET signaling is critical for the self-renewal and proliferation of SSCs [21]. Due to the importance that GDNF demonstrated *in vivo* and *in vitro* studies in the past several years, GDNF has been viewed as an indispensable factor for the long culture of SSCs to maintain their proliferation and self-renewal *in vitro* in murine models [43, 44]. However, there is little information on the effect of GDNF on the expansion of goat SSCs. Our previous study showed that GDNF could maintain goat SSC self-renewal and that GDNF up-regulated c-Myc expression via the PI3K/Akt pathway to promote goat SSC proliferation [45]. In this study. we also found that in GDNF deficient SSC medium *in vitro*, the SSC colony was almost lost. Meanwhile, we combined GDNF with melatonin to culture goat SSCs, and the results of SSC colony formation and the relative protein or mRNA expression levels showed an obvious increase in goat SSC proliferation compared with the GDNF(−) medium. These results indicate that the GDNF deficiency induced by the loss of goat SSC proliferation can be offset by melatonin, further illustrating that melatonin can promote Sertoli cell produced GDNF. The increased AKT phosphorylation levels indicated that the downstream GDNF-GFRa1-RET pathway was activated (Figure 7). This suggests that melatonin promotes SSC proliferation through the GDNF-GFRa1-RET-PI3K-AKT pathway. Interestingly, we also found that the ERK1/2 phosphorylation levels were increased. In the previous study, we found that the Ras/ERK1/2 pathway plays a critical role in maintaining the self-renewal of dairy goat SSCs via regulating ETV5 and BCL6B [46, 47]. Studies showed that FGF2, another factor secreted by Sertoli cells, may regulate mouse SSC proliferation and stem cell activity *in vitro* via phosphorylation of the AKT and ERK1/2 pathways [46, 47]. Thus we speculated that melatonin might also influence the secretion of FGF2; further studies will focus on FGF2.

In our study, the effect of melatonin on SSCs culture *in vitro* was not concentration-dependent and was contrary to seasonal breeding. The results may be attributed to mammalian reproduction being regulated by many factors, such as hormones and the nervous system [48, 49]. In males, melatonin affects reproductive regulation through the secretion of Gonadotropin-releasing hormone (GnRH) and Luteinizing hormone (LH), testosterone synthesis, and testicular maturation [48]. In this study, we found for the first time that the regulation of melatonin on goat Sertoli cells and SSCs may only be part of the reproduction regulation network, and our results provide a novel method of culturing SSCs *in vitro.*

Taken together, we found that melatonin can increase the secretion of GDNF in Sertoli cells. GDNF binds GFRa1-RET, leading to the phosphorylation of the AKT and ERK pathways to regulate the proliferation and self-renewal of dairy goat SSCs. Our findings may provide a new model to understand the mechanism of melatonin on mammal reproduction.

## MATERIALS AND METHODS

### Testes collection

Male dairy goat testes at different ages (6 month and 12 month) were collected from a local slaughterhouse and transported to the lab within 1 h in April and December. All of the procedures were conducted under the Guideline and Regulation of Chinese Experimental Animal Society.

### Isolation of testicular cells

Guanzhong dairy goat testes were washed 5–10 times with phosphate buffered saline (PBS) supplemented with 100 IU/ml penicillin and 100 IU/ml streptomycin. The seminiferous tubules were stripped from each testis and then dissected into small pieces using forceps. Seminiferous epithelial cells were dissociated using 2 mg/ml collagenase IV (Gibco, Carlsbad, CA) and 20 μg/ml DNase (Sigma, St. Louis, MO). The digestion was conducted at 37 °C for 15 min with pipetting performed every 5 min for the first step. After centrifugation at 2000 rpm for 10 min, the fragments of the seminiferous tubules were collected and digested with TD (0.25% trypsin(GIBCO, Carlsbad, CA) and 10 μg/ml DNaseI (Roche, Basilea, Swiss) for 10 min at 37 °C. The supernatant was processed by filtration through a 60-μm nylon mesh after centrifugation at 1500 rpm for 5 min [24].

### Cell culture

Undifferentiated SSCs were enriched using a differential attachment technique. Briefly, the cell suspension was placed into a gelatin-coated culture dish and incubated for 4 h at 37°C. The un-adherent cells were transferred into 12-well plates. SSCs were cultured in Dulbecco's modified Eagle's medium (DMEM/F12, GIBCO, Carlsbad, CA) supplemented with 1% fetal bovine serum (FBS, GIBCO), 10% Knockout serum replacement (KSR, Invitrogen, Carlsbad, CA) medium, 4 mM L-glutamine (Invitrogen), 1% non-essential amino acids (Invitrogen), 100 IU/ml penicillin, 100 IU/ml streptomycin (Invitrogen), 10 ng/ml GDNF, 10 ng/ml epidermal growth factor (EGF), 10 ng/ml basic fibroblast growth factor (bFGF), and 20 ng/ml glial cell line derived neurotrophic factor family receptor alpha 1 (GFRa1) [25]. The cells were cultured at 37 °C in a humidified atmosphere with 5 % CO2 for 3 passages. GDNF and melatonin (Sigma, St. Louis, MO) were then supplied according to the group design for 48 h.

### Real-time quantitative PCR (QRT-PCR)

Total RNA was extracted from goat testisand cultured SSCs using RNAiso (TaKaRa, Biotech. Co. Ltd., Dalian, China). Total RNA was reverse transcribed into cDNAs using the Reverse Transcriptase reagent kit according to the manufacturer's instructions (Thermo Scientific, FL33407, USA). QRT-PCR was performed on a CFX96 QRT-PCR detection system (Bio-Rad, CA 94547, USA) according to the instructions for the BioEasy SYBR Green I RT-qPCR kit (Bioer Co. Ltd., Hangzhou, China). The QRT-PCR was performed as described previously [26]. The relative expression levels of the target genes were normalized to *Gapdh* expression for each sample. The relative expression levels were calculated using 2^−ΔΔCt^. The primers for the validated mRNAs are listed in S2 Text.

### Western blot

The cultured SSCs were digested by RIPA (Beyotime, ShangHai, China) at 4 °C for 30 min and the protein were degenerated in 5×SDS sample loading buffer at 100°C for 10 min. Total protein was separated by SDS-PAGE 100V for 90 min, transferred to a 0.22-μm PVDF membrane at approximately 200 mA for 90 min, and incubated with B-ACTIN (1:1000, Beyotime, Shanghai, China), SOX9 (1:500, BOSTER, Wuhan, China), PCNA (1:1000, BOSTER), PLZF (1:300, Santa Cruz, USA), p-AKT (1:1000, Sangon Biotech, Beijing, China), AKT (1:1000, Sangon Biotech), ERK (1:1000, CST, Beverly, MA, USA), p-ERK (1:1000, CST). Horseradish peroxidase-conjugated anti-rabbit or anti-mouse were used as a secondary antibody (1:2000, BOSTER). Detection was performed using the Thermo Scientific Pierce ECL western blot substrate (Thermo Scientific, USA). The results were analyzed with a Tanon-410 automatic gel imaging system (Tanon Corporation, Shanghai, China).

### Testicular tissue immunohistochemistry and hematoxylin-eosin staining

Dairy goat testes were fixed in 4% formaldehyde overnight, dehydrated through a series of graded alcohols, and embedded in paraffin at 65°C for 6–8 h. The paraffin was then sectioned at 2 μm. The following step was performed as described previously [27]. The primary antibodies MT1(1:100, BOISS, Beijing, China) and MT2(1:100, BOISS, China) were incubated at 4°C overnight and then DBA (Beijing Zhongshan Golden Bridge Biochemical Factory, Beijing, China) was added and incubated at room temperature for 3 min. For Hematoxylin-eosin staining, the sections were cut at 5 μm and stained with Hematoxylin & Eosin staining.

### ELISA

The medium for measuring the GDNF levels was collected after culturing goat SSCs for 48 h. The medium was frozen at −80 °C until analysis. GDNF levels were determined by using a Human glial cell line-derived neurotrophic factor (GDNF) ELISA Kit (BOSTER). Briefly, 100 μl of standard and sample were added per well in 96-well plates (12 × 8 coated Microwell) and incubated for 90 min at 37°C. Removed the liquid of each well and added 100 μl of Biotin-antibody to each well, and incubated for 90 min at 37°C. Each well was aspirated and washed 3 times with washing Buffer. ABC Buffer was added and incubated for 30 min at 37°C and washed 5 times. Finally, TMB was added and the GDNF levels were determined by absorbance at 450 nm. The standard curve indicated a direct relationship between optical density and GDNF concentration.

### CCK-8

A total of 5*10^3^ cultured cells were plated into 96-well plates, with five parallel wells in each group. After a 48 h culture, the cell densities were determined using a CCK-8 assay kit (Beyotime, Shanghai, China), following the instructions provided by the manufacturer.

### Statistical analysis

The data are presented as the mean ± SEM. Differences in the expression of specific markers were evaluated using Student's t-test (Excel, Microsoft Corporation, USA). The results of the different treatments were considered statistically significant at P < 0.05 and were considered highly significant at P<0.01.

## SUPPLEMENTARY MATERIALS FIGURE AND TABLE



## Abbreviations

SSCs: spermatogonia stem cells
GDNF: Glial cell line–derived neurotrophic factor
NSCs: neural stem cells
MSCs: mesenchymal stem cells
iPSCs: induced pluripotent stem cells
ESCs: embryonic stem cells
QRT-PCR: Real-time quantitative PCR
AP: alkaline phosphatase
MT: melatonin receptor
BDNF: Brain-Derived Neurotrophic Factor
GFRA1: GDNF family receptor alpha-1
ELISA: Enzyme-linked immunosorbent assay
FBS: fetal bovine serum
KSR: Knockout serum replacement
EGF: epidermal growth factor
bFGF: basic fibroblast growth factor
TGF–β: transforming growth factor–β
GnRH: Gonadotropin-releasing hormone
LH: Luteinizing hormone.
