# Supplementary material for: Melatonin promotes goat spermatogonia stem cells (SSCs) proliferation by stimulating glial cell line-derived neurotrophic factor (GDNF) production in Sertoli cells
Source: Oncotarget. 2016 Oct 18;7(47):77532–42. doi: 10.18632/oncotarget.12720 (PMC5363602; doi:10.18632/oncotarget.12720)
Supplement: Supplementary file 1 [file oncotarget-07-77532-s001.pdf]

Melatonin promotes goat spermatogonia stem cells (SSCs) proliferation by stimulating glial cell line-derived neurotrophic factor (GDNF) production in Sertoli cells

SUPPLEMENTARY FIGURE AND TABLE

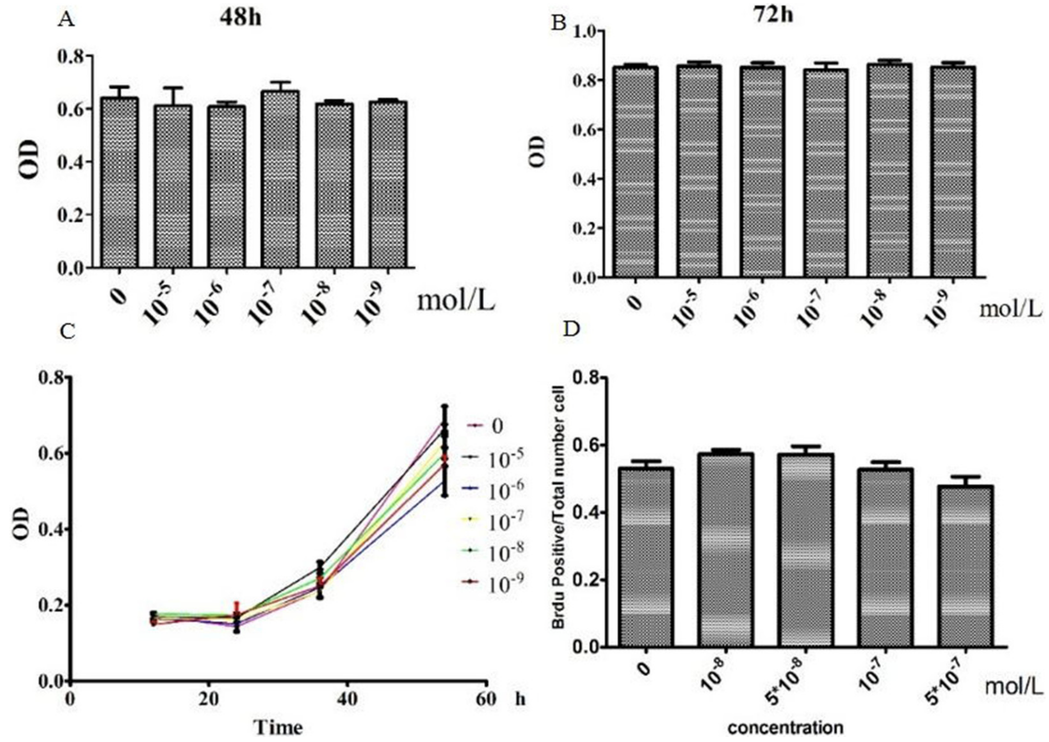

Supplementary Figure S1: Analysis the proliferation of mGSCs-I-SB affected by melatonin. A. CCK-8 OD value after treated with melatonin 48 hours. **B.** CCK-8 OD value after treated with melatonin 72 hours. **C.** MTT OD value after treat with melatonin 12, 24, 36, 55 hours. **D.** Brdu positive cell rate after treated with melatonin 48hours.

Supplementary Table S1: Primers used for QRT-PCR

Gene	Primer		Tm (°C)	
	Forward	Reverse		
Gfra1	CGCCTTGCGGATTTTTTTACC	ACACGGTCACATCGGAGCCA	286	58
Plzf	CACCGCAACAGCCAGCACTAT	CAGCGTACAGCAGGTCATCCAG	127	58
Pcna	AGTGGAGAACTTGGAAATGGAA	GAGACAGTGGAGTGGCTTTTGT	185	58
Sox9	ACCGCCTTGTCGTTAGACCG	TCCACGCTCGCTTTGAAGGT	101	58
Gdnf	GCAGCCGAAACAATGTACGA	AAGGCGATGGGTCTGCAA	101	60
CyclinA	TGGCTGTGAACTACATTGA	ACAAACTCTGCTACTTCTGG	136	59
Etv5	CAGTGTTCTCGGAGCGGTT	AGCAATTTCTCGGGGGAAGG	146	58
Stra8	CATCTCCCATCTGTGGCA	TAGAGATGAAACTTGTCCTC	212	58
Sycp3	GTATGGAGGACTTGGAGA	GAGACTTTCGGACACTTGC	138	58
Kit	TCCCAAACCTCAACACCGACAG	GTGTAAGTGCCTCCTTCAGTCCC	153	58
Dazl	CAAGTTCACCAGTTCAGGTTATCAC	GACAACGGAGTTTCTCAGTCTATTC	190	58
Gapdh	CGGCTCTCAAGGGCATTCTAGGC	TGAGGTCCACCACCCTGTTGCTG	182	58
